# *Capsicum annuum* Regulates Tumor Growth Through Modulation of TLR4/PI3K Signaling in a Lewis Lung Carcinoma Mouse Model

**DOI:** 10.3390/antiox15070871

**Published:** 2026-07-13

**Authors:** Hye Ji Choi, Hyo Lim Lee, Yeong Hyeon Ju, Yu Mi Heo, Hwa Rang Na, Chae Eun Yoon, Young Hee Son, Do-Yoon Kim, Yu-Jin Kim, Hui-Seok Jeong, Seung-Hwan Park, Hyun-Jin Kim, Ho Jin Heo

**Affiliations:** 1 Division of Applied Life Science (BK21), Institute of Agriculture and Life Science, Gyeongsang National University, Jinju 52828, Republic of Korea; hjchoi0820@gnu.ac.kr (H.J.C.); gyfla059@gnu.ac.kr (H.L.L.); ju8172001@gnu.ac.kr (Y.H.J.); yumi@gnu.ac.kr (Y.M.H.); hrna@gnu.ac.kr (H.R.N.); codmsy0114@gnu.ac.kr (C.E.Y.); yhson@gnu.ac.kr (Y.H.S.); ehdbs0228@gnu.ac.kr (D.-Y.K.); yujinkim@gnu.ac.kr (Y.-J.K.); 2025210068@gnu.ac.kr (H.-S.J.); hyunjkim@gnu.ac.kr (H.-J.K.); 2 Agriculture Research Center for Carbon Neutral and Healing, Gurye 57607, Republic of Korea; red42co@hanmail.net

**Keywords:** lung cancer, *Capsicum annuum*, carotenoids, TLR4/NF-κB, PI3K/Akt/mTOR

## Abstract

Since lung cancer remains a leading cause of cancer-related mortality, effective adjunct strategies against this disease are needed. This study evaluated an extract prepared from organically cultivated *Capsicum annuum* treated with deep-seawater-derived ion minerals during cultivation (ODSW-CE) in Lewis lung carcinoma (LLC1) cells and LLC1 tumor-bearing mice. Targeted HPLC-DAD analysis identified and quantified lutein and β-carotene as representative carotenoids. ODSW-CE reduced MTT-derived cell viability and colony-forming ability in vitro and suppressed tumor growth in vivo. In tumor-bearing mice, ODSW-CE was associated with lower hematological inflammatory indices, reduced TNF-α and IL-1β levels in plasma and lung tissue, and attenuated histopathological alterations in tumor and lung tissues. These changes were accompanied by reduced expression of proteins associated with TLR4/MyD88/NF-κB signaling. ODSW-CE also modulated survival and apoptosis-related proteins, decreasing PI3K, p-Akt, and p-mTOR expression, increasing Bax and cleaved caspase-3 expression, and decreasing Bcl-2 expression. Additionally, in tumor tissue, Keap1 expression increased, whereas Nrf2, xCT, and GPX4 expression decreased. Overall, ODSW-CE suppressed LLC1 tumor growth and was associated with coordinated modulation of tumor-associated inflammatory signaling, survival pathways, apoptosis-related proteins, and redox defense.

## 1. Introduction

Associated with high incidence and mortality worldwide [1,2,3], lung cancer is often diagnosed at an advanced stage and is difficult to treat due to its high heterogeneity and metastatic propensity [4]. Due to these characteristics, surgical resection is accompanied by various treatment strategies, including cytotoxic chemotherapy, targeted therapies, and immunotherapy in clinical practice [2]. Among the cytotoxic agents used in lung cancer treatment, cisplatin is a representative platinum-based drug that inhibits cancer cell proliferation by binding to DNA and interfering with its replication and transcription [5]. Cisplatin affects normal tissues by potentially causing side effects, such as neurotoxicity, nephrotoxicity, and myelosuppression [5]. Similarly, other lung cancer treatment modalities are also limited by distinct adverse effects, treatment resistance, incomplete response, and recurrence [2,6]. To complement the limitations of treatment, a new treatment approach must simultaneously control multiple pathophysiological axes supporting tumor progression [2].

Given these limitations, natural product-derived materials that can simultaneously modulate multiple pathophysiological axes have gained increasing attention [7]. The progression of lung cancer involves not only the intrinsic properties of tumor cells but also the coordinated interplay of immune and inflammatory responses, tumor cell survival signaling, and redox homeostasis within the tumor microenvironment [3,7]. Under hypoxic conditions, metabolic reprogramming, necrosis, and tissue damage accumulate in the tumor microenvironment due to rapid tumor growth [8]. Reactive oxygen species (ROS) levels increase during this process, with the accumulation of danger signals originating from damaged cells and remodeled tissues [9,10]. These signals stimulate Toll-like receptor (TLR) 4, which leads to myeloid differentiation primary response 88 (MyD88)-dependent nuclear factor-kappa B (NF-κB) activation. This further enhances tumor-promoting inflammatory responses [3,4]. This inflammatory and oxidative microenvironment can be linked to the activation of the phosphoinositide 3-kinase (PI3K)/protein kinase B (Akt)/mechanistic target of rapamycin (mTOR) axis, which supports tumor cell growth and survival [6,11]. Increased ROS may further amplify inflammatory and survival signals [9]. At the same time, cancer cells activate antioxidant and redox defense systems to adapt to this oxidative stress [9,12]. Therefore, tumor progression in lung cancer can be understood as a process in which immune and inflammatory responses, tumor cell survival signals, and changes in redox homeostasis interact. There is a growing interest in physiologically active materials of plants, which can modulate all these processes.

*Capsicum annuum* L., a member of the Solanaceae family, is widely consumed worldwide and contains diverse bioactive compounds, such as capsaicinoids, carotenoids, flavonoids, polyphenols, and vitamins [13]. Carotenoids, capsaicin, and dihydrocapsaicin have been reported to exhibit antioxidant, anti-inflammatory, antimicrobial, and anticancer activities [13,14]. Lutein, a carotenoid, suppresses inflammatory responses by regulating the TLR4/nucleotide-binding oligomerization domain-like receptor family pyrin domain-containing protein 3 (NLRP3)/caspase-1-related signaling [15,16]. Beta carotene (β-carotene) alleviates oxidative stress and inflammation-related dysfunction in pathological conditions [17,18]. The content and composition of these bioactive compounds may vary depending on the cultivation conditions [19]. Abiotic factors, such as light, temperature, water stress, elicitor treatment, and soil mineral composition, can influence the biosynthesis of bioactive compounds to alter their qualitative and quantitative profiles in pepper fruits [19]. Minerals derived from deep seawater (DSW) may influence the phytochemical profiles of peppers, which contain balanced levels of essential macro- and microelements [19,20,21]. On the basis of preliminary comparisons between the extracts of peppers cultivated conventionally and organically, we selected an organic deep-sea water mineral-treated chili pepper extract for investigation (Figures S1–S3). Accordingly, the objective of this study was to determine whether an extract prepared from organically cultivated *Capsicum annuum* treated with deep-seawater-derived ion minerals during cultivation suppresses the growth of Lewis lung carcinoma (LLC1) and whether its antitumor effects are associated with changes in tumor-promoting inflammatory and prosurvival signaling. To address this objective, we evaluated the antiproliferative activity of the extract in LLC1 cells and examined its effects on tumor growth and proteins related to inflammation, apoptosis, and redox defense in an LLC1 subcutaneous tumor mouse model.

## 2. Materials and Methods

### 2.1. Preparation of ODSW-CE

Chili pepper (*Capsicum annuum* L. cultivar ‘Mi-in’; Asia Seed Co., Seoul, Republic of Korea) was obtained from iCOOP Natural Dream Company (Gurye, Republic of Korea). The peppers were cultivated in a glass greenhouse research field of the Organic Anti-Cancer Agriculture Research Institute, located in Gurye, Republic of Korea. Whole fruits, including the seeds but excluding the peduncles, were used in this study.

The peppers were grown under certified organic conditions and additionally treated with a deep-seawater-derived ion-mineral solution during cultivation. Beginning after the onset of flowering, the ion-mineral solution, diluted 2000-fold, was applied by soil drenching once weekly. In contrast, the standard organic cultivation condition did not include this additional ion-mineral treatment. In this study, the extract prepared from organically cultivated *C. annuum* subjected to the ion-mineral treatment was designated ODSW-CE. Thus, “ODSW” refers to the cultivation condition of the source plant material, and the ion-mineral solution was not used as an extraction solvent. The fertilizers used in this cultivation system contained multiple mineral components, including Na, Mg, Ca, K, Fe, phosphate, Si, Cl, N, and B [21]. Additional details regarding the cultivation system have been reported previously [20,22].

The harvested whole pepper fruits were freeze-dried and ground into powder prior to extraction. For carotenoid extraction, 220 g of chili pepper powder was extracted with 880 mL of hexane for 12 h. The extract was centrifuged at 710× *g* for 5 min at 4 °C, and the resulting supernatant was mixed with 2 L of 10% (*w*/*v*) sodium chloride solution. The mixture was centrifuged again under the same conditions to separate the organic and aqueous phases. The upper organic phase was collected and concentrated to dryness using a centrifugal vacuum concentrator (Hyper-VAC VC2124; Hanil Scientific Inc., Gimpo, Republic of Korea). The dried residue was reconstituted in 200 μL of methyl *tert*-butyl ether (MTBE):methanol (1:1, *v*/*v*), and the supernatant obtained after centrifugation was collected as a crude carotenoid-rich extract from chili peppers. This extract was used for subsequent in vitro and in vivo experiments.

### 2.2. Determination of Carotenoid Content

Carotenoids in the extract, including lutein (Sigma-Aldrich Chemical Co., St. Louis, MO, USA; 07168) and β-carotene (Sigma-Aldrich Chemical Co., St. Louis, MO, USA; 222040), were analyzed by HPLC-DAD (Shimadzu Corp., Tokyo, Japan) using a carotenoid column (250 mm × 4.6 mm i.d., 5 µm; YMC Co., Ltd., Kyoto, Japan). The column temperature was set at 40 °C. The mobile phases consisted of solvent A (MeOH:MTBE:distilled water = 85:10:5, *v*/*v*/*v*) and solvent B (100% MTBE). The flow rate was 0.8 mL/min, the injection volume was 10 μL, and detection was performed at 450 nm. The gradient program was as follows: 30% B at 0 min, held until 1 min, increased linearly to 100% B by 15 min, held at 100% B until 17 min, returned to 30% B at 18 min, and held until 22 min. Calibration curves were generated using lutein (5–100 μg/mL) and β-carotene (6.25–100 μg/mL) standard solutions. Carotenoids were identified by comparing retention times and ultraviolet (UV)-visible (Vis) absorption spectra with those of authentic standards and quantified based on peak area. Additional characterization of the lipophilic constituents of ODSW-CE was performed by ultra-performance liquid chromatography (UPLC)-DAD-atmospheric chemical ionization (APCI)-quadrupole time-of-flight mass spectrometer (QTOF/MS). The complete analytical procedure is provided in Supplementary Information.

### 2.3. Cell Study

#### 2.3.1. Cell Culture

LLC1 cells were purchased from the American Type Culture Collection (ATCC, Manassas, VA, USA; CRL-1642) and maintained in Dulbecco’s Modified Eagle’s Medium, high glucose (DMEM; Sigma-Aldrich Chemical Co., Milwaukee, WI, USA; D6429) supplemented with 10% fetal bovine serum (FBS; Corning, Madison, NY, USA; 35-015-CV) and 1% penicillin–streptomycin solution (Sigma-Aldrich Chemical Co., Milwaukee, WI, USA; P4333). Cells were cultured at 37 °C in a humidified atmosphere containing 5% CO_2_. Subculturing was performed when cells reached approximately 80% confluence in T-flasks (SPL Life Sciences, Pocheon, Republic of Korea; 70075).

#### 2.3.2. Cell Viability Assay

LLC1 cells were seeded at 5 × 10^3^ cells/well in 96-well plates (SPL Life Sciences, Pocheon, Republic of Korea; 30096) and incubated for 24 h to allow attachment. Cells were treated with ODSW-CE (50–1000 μg/mL) and incubated for an additional 24, 48, and 72 h. The 3-(4,5-dimethylthiazole-2-yl)-2,5-diphenyl tetrazolium bromide (MTT) solution (5 mg/mL; Alfa Aesar, Haverhill, MA, USA; L11939) was then added to each well, and plates were incubated at 37 °C for 3 h. The medium was aspirated, and formazan crystals were dissolved in dimethyl sulfoxide (DMSO; DAEJUNG Chemicals & Metals Co., Ltd., Gyeonggi-do, Republic of Korea; 3047-4100). Absorbance was measured at 570 nm (reference, 630 nm) using a microplate reader (Epoch 2; Bio-Tek Instruments, Inc., Winooski, VT, USA). The inhibition rate of each well was calculated relative to the mean absorbance of the untreated control group using the following equation:

(1) $$\mathrm{Inhibition}\;\mathrm{Rate}\;(\%)=1-\frac{Absorbance\;of\;each\;well}{Mean\;absorbance\;of\;control\;group}\times 100$$

#### 2.3.3. Colony Formation Assay

LLC1 cells were seeded at 5 × 10^2^ cells/well in 6-well plates (SPL Life Sciences, Pocheon, Republic of Korea; 30006). After 24 h, once cells had adhered, ODSW-CE was treated (50–1000 μg/mL). Cells were cultured for approximately 2 weeks until discrete colonies became visible. The medium was removed, and cells were washed with phosphate-buffered saline (PBS), fixed with 100% methanol for 10 min, and washed again with PBS. Colonies were stained with crystal violet solution (Sigma-Aldrich Chemical Co., St. Louis, MO, USA; C0775) for 20 min and rinsed with distilled water. Colonies were defined as cell clusters containing ≥50 cells and quantified using ImageJ software (version 1.54, NIH, Bethesda, MD, USA). The colony count of each replicate was expressed relative to the mean colony count of the control group, which was set to 100%.

### 2.4. Animal Study

#### 2.4.1. Animal Design

The Institutional Animal Care and Use Committee (IACUC) of Gyeongsang National University (GNU-250711-M0156, date of approval: 11 July 2025) approved all the animal experiments. Male C57BL/6 mice, which were six weeks old, were purchased from Samtako (Samtako BIO KOREA Co., Ltd., Osan, Republic of Korea) and housed under controlled conditions (temperature: 25 ± 2 °C, relative humidity: 55 ± 10%, 12 h light/dark cycle), with ad libitum access to water and standard chow. After a one-week acclimatization period, the mice were randomly allocated into five groups (*n* = 7 per group): Normal Control (NC), Tumor, ODSW-CE low-dose group (ODSW-CE-L; 100 mg/kg body weight/day), ODSW-CE high-dose group (ODSW-CE-H; 200 mg/kg body weight/day), and positive control (cisplatin).

Except for the mice in the NC group, the other mice received a subcutaneous injection of 1 × 10^6^ LLC1 cells suspended in cold PBS in the right flank. The NC group received an equivalent volume of cold PBS. After 24 h, the NC and Tumor groups received corn oil by oral gavage once daily for 21 consecutive days (Sigma-Aldrich Chemical Co., St. Louis, MO, USA; C8267). The ODSW-CE-L and ODSW-CE-H groups received ODSW-CE dissolved in corn oil at 100 and 200 mg/kg body weight/day, respectively, by oral gavage once daily for 21 consecutive days. The cisplatin group received intraperitoneal injections of cisplatin (4 mg/kg body weight; Sigma-Aldrich Chemical Co., St. Louis, MO, USA; P4394) dissolved in saline every 3 days. After the 21-day treatment period, all animals were euthanized by CO_2_ inhalation.

Every three days, the body weight and tumor dimensions of the mice were recorded. Tumor volume was calculated as follows:

(2) $$\mathrm{Tumor}\;\mathrm{Volume}\;({\mathrm{mm}}^{3})=\frac{length\;\left(mm\right)\times width\;({mm}^{2})}{2}$$

#### 2.4.2. White Blood Cell Analysis

Whole blood collected at sacrifice was placed into K_2_EDTA tubes (BD Biosciences, Franklin Lakes, NJ, USA) and analyzed using a SYSMEX XN-V automated hematology analyzer (Sysmex Corporation, Kobe, Japan). Differential white blood cell counts, including neutrophils, lymphocytes, and monocytes, were obtained. The neutrophil-to-lymphocyte ratio (NLR) and monocyte-to-lymphocyte ratio (MLR) were calculated as follows:

(3) $$\mathrm{NLR}=\frac{Neutrophil\;count}{Lymphocyte\;count}$$

(4) $$\mathrm{MLR}=\frac{Monocyte\;count}{Lymphocyte\;count}$$

#### 2.4.3. Enzyme-Linked Immunosorbent Assay (ELISA)

Blood collected at sacrifice was transferred to heparin tubes (Fujifilm, Tokyo, Japan) and centrifuged (LaboGene, Daejeon, Republic of Korea) at 10,000× *g* for 10 min at 4 °C to isolate plasma. The plasma supernatant was collected and stored at 4 °C until analysis. TNF-α and IL-1β concentrations in plasma and lung tissue were determined using commercially available ELISA kits (KE10002, KE10003, Proteintech, Rosemont, IL, USA) according to the manufacturer’s instructions. Cytokine concentrations in plasma were expressed as pg/mL. Lung homogenate cytokine concentrations were normalized to the total protein content of each sample as determined by the Bradford protein assay and expressed as pg/mg of protein.

#### 2.4.4. Hematoxylin and Eosin (H&E) Staining

To evaluate histopathological changes, the left lung lobe and tumor tissue were fixed in 10% neutral buffered formalin (Sigma-Aldrich Chemical Co., St. Louis, MO, USA; HT501128). Fixed tissues were embedded in paraffin and sectioned at 4 μm. H&E-stained slides were digitally scanned using a Motic EasyScan Pro 6 whole-slide scanner (Motic, Hong Kong, China).

#### 2.4.5. Western Blot Analysis

Tumor tissues were homogenized in cell extraction buffer (GeneAll Biotechnology, Seoul, Republic of Korea; 701-001) containing 1% protease inhibitor (Quartett, Berlin, Germany; QTPPI1015) at a 1:10 (*w*/*v*) ratio. Homogenates were centrifuged at 13,000× *g* for 15 min at 4 °C, and the supernatants were collected. Protein concentrations were determined using the Bradford assay (Bio-Rad Laboratories, Inc., Hercules, CA, USA; 5000006). Samples were prepared by adding 4× sample buffer (Bio-Rad Laboratories, Inc., Hercules, CA, USA; 1610747) and heating at 95 °C for 10 min. Equal amounts of protein were resolved by SDS-PAGE on 6–12% polyacrylamide gels and transferred onto PVDF membranes (Millipore, Billerica, MA, USA; IPVH00010). Membranes were blocked with 5% skim milk (MBcell, Seoul, Republic of Korea; MB-S1667) for 1 h at room temperature and incubated overnight at 4 °C with primary antibodies (1:1000; Table 1). After washing, membranes were incubated with secondary antibodies (1:3000; Table 1) for 1 h at room temperature. Protein bands were visualized using an enhanced chemiluminescence reagent (ECL; TransLab, Seoul, Republic of Korea; TLP-112.1) and detected using an iBright™ CL1000 imaging system (Invitrogen, Carlsbad, CA, USA). Band intensities were quantified using ImageJ software (version 1.54; NIH, Bethesda, MD, USA). For each mouse, the intensity of each target protein was divided by the β-actin intensity from the same sample. For each protein, the resulting target/β-actin ratios were then expressed relative to the mean ratio of the Tumor group, which was set to 1.0.

### 2.5. Statistical Analysis

Statistical analyses and graph preparation were performed using GraphPad Prism 10 (GraphPad Software, Boston, MA, USA). Data are presented as the mean ± SD. Normality was assessed using the Shapiro–Wilk test, and no datasets significantly deviated from normality. Accordingly, group differences were evaluated by one-way analysis of variance (ANOVA) followed by Tukey’s multiple-comparisons test. Differences were considered statistically significant at *p* < 0.05.

## 3. Results

### 3.1. HPLC Quantification of Lutein and β-Carotene in ODSW-CE

**Figure 1:**
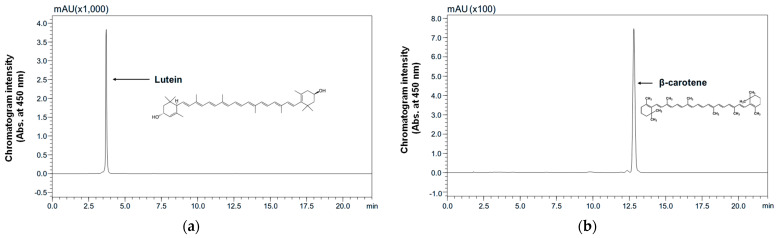

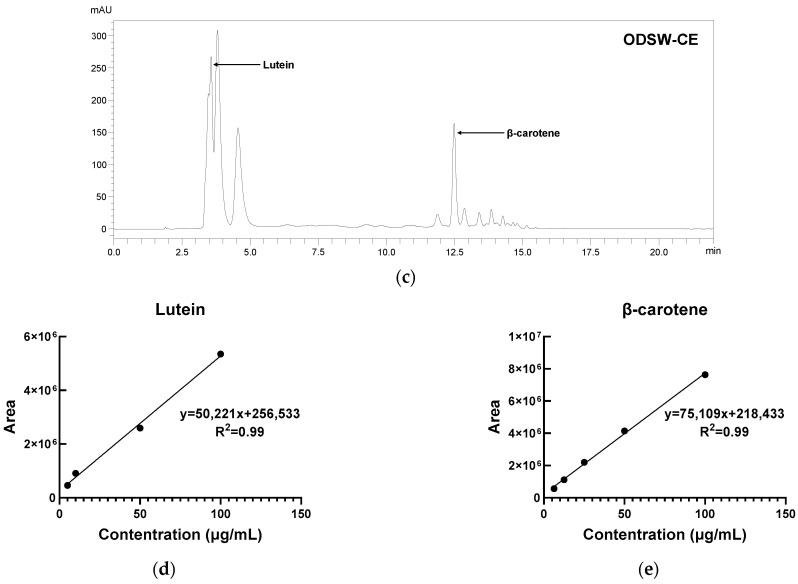
**Quantitative analysis of lutein and β-carotene in ODSW-CE. Representative** chromatograms of the lutein standard (**a**), β-carotene standard (**b**), and ODSW-CE (**c**) recorded by high-performance liquid chromatography (HPLC)–diode array detection (DAD) at 450 nm. Calibration curves for lutein (**d**) and β-carotene (**e**).

Targeted HPLC-DAD analysis was performed using authentic lutein and β-carotene standards (Figure 1a). Peaks corresponding to lutein and β-carotene were assigned by comparing their retention times with those of the respective standards. The lutein standard eluted at 3.83 min, and a corresponding peak was detected in ODSW-CE (3.55 min) (Figure 1b). Similarly, the β-carotene standard eluted at 12.85 min, and a corresponding peak was observed in ODSW-CE at 12.46 min (Figure 1c). External calibration curves were prepared over concentration ranges of 5–100 μg/mL for lutein and 6.25–100 μg/mL for β-carotene, yielding regression equations of y = 50,221x + 256,533 (R^2^ = 0.99) and y = 75,109x + 218,433 (R^2^ = 0.99), respectively. Based on these calibration curves, the contents of lutein and β-carotene were 3.65 ± 0.11 and 0.81 ± 0.01 mg/100 g dry weight of *C. annuum*, respectively. Several additional chromatographic peaks remained unassigned by the targeted HPLC-DAD analysis. Exploratory UPLC-DAD-APCI-QTOF/MS analysis was therefore performed to further characterize these constituents. Several features were putatively annotated as carotenoid-related compounds based on accurate-mass measurements and comparison of their fragmentation patterns with published data. The corresponding DAD chromatogram and putative compound annotations are presented in Figure S4 and Table S1, respectively.

### 3.2. Anti-Proliferative Effects of ODSW-CE

**Figure 2:**
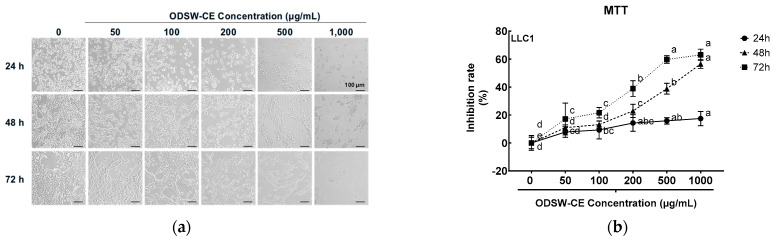

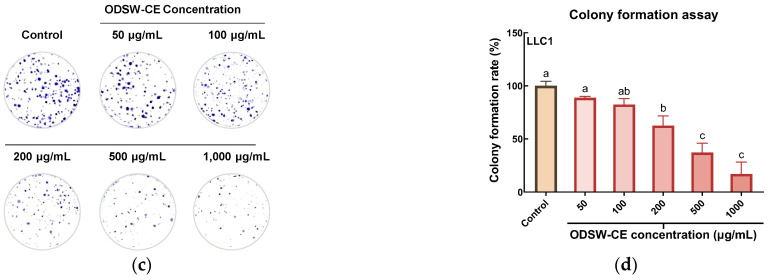
Cell morphology changes of the LLC1 cells treated with ODSW-CE, black line indicates scale bar = 100 μm (**a**). Time- and dose-dependent effects of the ODSW-CE on LLC1 cell viability at 24, 48, and 72 h, assessed by MTT assay (**b**). Representative images (**c**) and quantification of colony formation assay in LLC1 cells treated with the ODSW-CE for 14 days (**d**). Data are expressed as mean ± SD (*n* = 3). In subfigures (**b**,**d**), different lowercase letters indicate significant differences among groups (*p* < 0.05) as determined by one-way ANOVA followed by Tukey’s multiple comparison test.

To evaluate the effects of ODSW-CE on LLC1 cell growth and proliferative capacity, we performed concentration- and time-dependent MTT assays and a colony formation assay (Figure 2). Morphological observation showed that cell density decreased as the ODSW-CE concentration increased (Figure 2a). Consistently, ODSW-CE reduced MTT-derived cellular metabolic activity in a concentration-dependent manner, and the magnitude of this reduction increased with longer exposure times (24, 48, and 72 h) (Figure 2b). The MTT-derived IC_50_ values at 48 and 72 h were 746.89 ± 25.11 μg/mL and 497.68 ± 37.39 μg/mL, respectively.

In the colony formation assay, both colony number and colony size decreased as the ODSW-CE concentration increased (Figure 2c). Quantification confirmed concentration-dependent inhibition of colony formation (Figure 2d).

### 3.3. ODSW-CE Attenuates Tumor Growth in a Subcutaneous Lewis Lung Carcinoma Model

**Figure 3:**
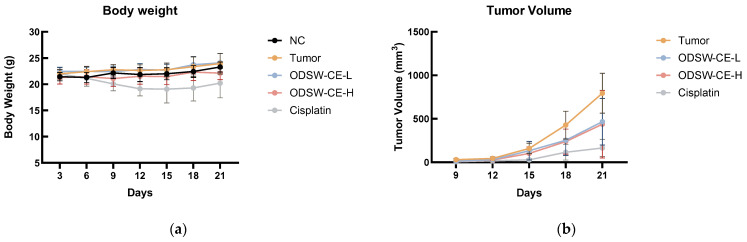

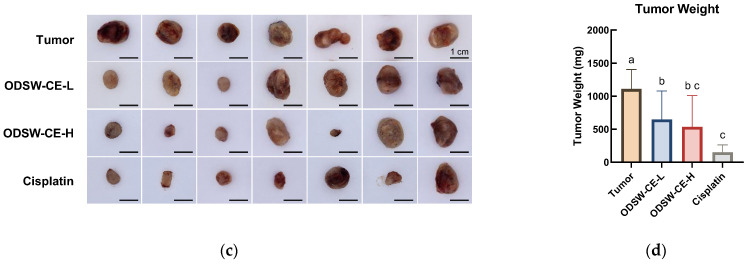
Anti-tumor effects of the ODSW-CE in the LLC1 transplanted mouse model. Body weight changes of mice in each group during the experimental period (**a**). Tumor volume (**b**). The morphology of a subcutaneous tumor in C57BL/6 mice, black line indicates scale bar = 1 cm (**c**). Tumor weight (**d**). Data are expressed as mean ± SD (*n* = 7). In subfigure (**d**), different lowercase letters indicate significant differences among groups (*p* < 0.05) as determined by one-way ANOVA followed by Tukey’s multiple comparison test.

To verify the anticancer effect of ODSW-CE, LLC1 cells were injected subcutaneously into C57BL/6 mice to induce tumors, followed by oral administration of ODSW-CE for 21 days. Body weight showed an overall increasing trend throughout the experimental period. In the cisplatin-treated positive control group, body weight was lower than that in the NC group, though this was not statistically significant (Figure 3a).

Tumor volume showed the greatest increase over time in the Tumor group, while the ODSW-CE-L and ODSW-CE-H treatment groups exhibited slower tumor growth compared with the Tumor group (Figure 3b). The cisplatin group demonstrated the slowest tumor growth (Figure 3c). Images of the excised tumors after the experiment are presented in Figure 3c. Measurement of tumor weight revealed lower tumor weights in the ODSW-CE-L group (646.66 ± 432.34 mg) and ODSW-CE-H group (534.90 ± 474.18 mg) compared with the Tumor group (1108.51 ± 294.04 mg), with the cisplatin group exhibiting the lowest tumor weight at 150.59 ± 111.31 mg (Figure 3d).

### 3.4. ODSW-CE Reduces Tumor-Associated Inflammatory Markers and Histopathological Alterations

**Figure 4:**
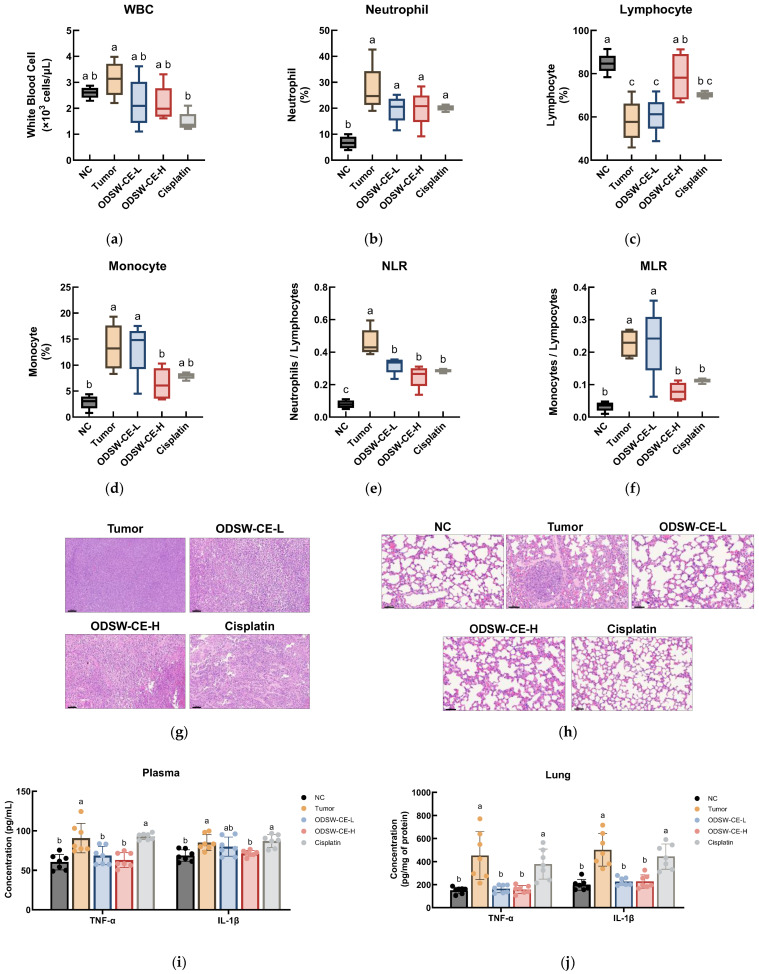
Effects of the ODSW-CE on hematological parameters, inflammatory cytokine levels, and histopathological changes in the LLC1-bearing mouse model. White blood cells (WBC) (**a**), neutrophil (**b**), lymphocyte (**c**), monocyte (**d**), neutrophil-to-lymphocyte ratio (NLR) (**e**), and monocyte-to-lymphocyte ratio (MLR) (**f**) in whole blood (*n* = 5). Hematoxylin and eosin (H&E)-stained sections of tumor tissues from each group (black line indicates scale bar = 100 μm) (*n* = 3) (**g**). H&E-stained sections of lung tissues from each group (black line indicates scale bar = 50 μm) (*n* = 3) (**h**). TNF-α and IL-1β levels in plasma were measured by ELISA (*n* = 7) (**i**). TNF-α and IL-1β levels in lung tissue were measured by ELISA (*n* = 7) (**j**). Data are presented as the mean ± SD. For each quantitative parameter, statistical comparisons were performed independently among groups. Groups with no lowercase letters in common are significantly different, whereas groups sharing at least one lowercase letter are not significantly different (*p* < 0.05), as determined by one-way ANOVA followed by Tukey’s multiple comparison test.

To assess tumor-associated inflammatory alterations, hematological indices were first analyzed (Figure 4a–f). Compared with the NC group, the Tumor group showed a higher neutrophil percentage and a lower lymphocyte percentage, together with increased monocyte levels, resulting in marked elevations of both the NLR and MLR. Total white blood cell counts tended to be higher in the Tumor group, although ODSW-CE treatment did not produce a clear reduction in this parameter. Likewise, neutrophil levels remained elevated across tumor-bearing groups. In contrast, ODSW-CE-H increased the lymphocyte percentage relative to the Tumor group, and both ODSW-CE-treated groups showed lower NLR values than the Tumor group. Monocyte levels were also lower in the ODSW-CE-H group than in the Tumor group, and this was accompanied by a reduction in MLR.

Histopathological examination further supported these findings (Figure 4g,h). Tumor sections from the Tumor group showed diffuse hypercellularity with densely packed tumor cells, whereas ODSW-CE-treated tumors exhibited reduced cellular density. This change was more evident in the ODSW-CE-H group, which showed broader acellular or hypocellular areas, similar to the cisplatin group. In lung tissue, the NC group showed intact alveolar architecture with well-preserved air spaces, whereas the Tumor group displayed inflammatory cell aggregates and disruption of alveolar structure. ODSW-CE treatment reduced inflammatory infiltration and improved preservation of alveolar architecture, with the effect again being more apparent in the ODSW-CE-H group.

To quantify systemic and local inflammatory mediators, TNF-α and IL-1β levels were measured in plasma and lung tissues (Figure 4i,j). In plasma, TNF-α levels were higher in the Tumor group and were lower in both ODSW-CE-treated groups. Plasma IL-1β showed a similar tendency, with a clearer reduction in the ODSW-CE-H group. In lung tissues, the protein-normalized levels of TNF-α and IL-1β were elevated in the Tumor group and reduced in the ODSW-CE-treated groups. Notably, these cytokine levels were lower in the ODSW-CE groups than in the cisplatin group, in which TNF-α and IL-1β remained relatively high.

### 3.5. ODSW-CE Inhibits TLR4/NF-κB-Mediated Inflammatory Signaling

**Figure 5:**
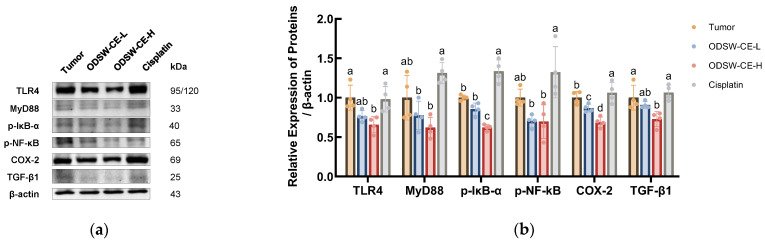
Effects of the ODSW-CE on the TLR4/MyD88/NF-κB signaling pathway in tumor tissues. Representative Western blot images showing the protein expression levels of TLR4, MyD88, p-IκB-α, p-NF-κB, COX-2, and TGF-β1 in tumor tissues from each group (**a**). Relative protein expression levels normalized to β-actin (**b**). Data are presented as the mean ± SD (*n* = 4). For each protein, statistical comparisons were performed independently among groups. Groups with no lowercase letters in common are significantly different, whereas groups sharing at least one lowercase letter are not significantly different (*p* < 0.05), as determined by one-way ANOVA followed by Tukey’s multiple comparison test.

The results of analyzing the expression of proteins related to TLR4/NF-κB signaling in tumor tissues are shown in Figure 5. In the ODSW-CE-H-treated group, the expression of TLR4 and MyD88 was lower than that in the Tumor group. The expression of p-IκB-α and p-NF-κB was also lower than that in the Tumor group. The expression of downstream factors, COX-2 and TGF-β1, was also lower in the ODSW-CE-H-treated group compared with the Tumor group. In the cisplatin-treated group, TLR4, MyD88, p-IκB-α, p-NF-κB, COX-2, and TGF-β1 levels were higher than those in the Tumor group.

### 3.6. ODSW-CE Suppresses PI3K/Akt/mTOR Signaling and Modulates Apoptosis-Related Proteins

**Figure 6:**
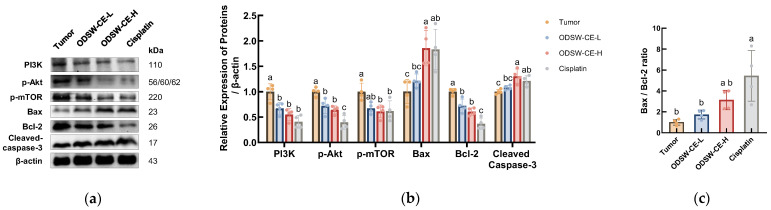
Effects of the ODSW-CE on the PI3K/Akt/mTOR signaling pathway and apoptosis-related proteins in tumor tissues. Representative Western blot images showing the protein expression levels of PI3K, p-Akt, p-mTOR, Bax, Bcl-2, and cleaved caspase-3 in tumor tissues from each group (**a**). Relative protein expression levels normalized to β-actin (**b**), and the Bax/Bcl-2 ratio (**c**). Data are presented as the mean ± SD (*n* = 4). For each protein, statistical comparisons were performed independently among groups. Groups with no lowercase letters in common are significantly different, whereas groups sharing at least one lowercase letter are not significantly different (*p* < 0.05), as determined by one-way ANOVA followed by Tukey’s multiple comparison test.

The results of analyzing the expression of PI3K/Akt/mTOR signaling proteins and apoptosis-related proteins in tumor tissues are shown in Figure 6. The ODSW-CE-H-treated group showed decreased expression of PI3K, p-Akt, and p-mTOR compared with the Tumor group. Furthermore, analysis of apoptosis-related proteins revealed that Bax expression was higher and Bcl-2 expression was lower in the ODSW-CE-H-treated group compared with the Tumor group. Also, the Bax/Bcl-2 ratio was higher in the ODSW-CE-treated group compared with the Tumor group. Cleaved caspase-3 expression was higher in the ODSW-CE-H-treated group compared with the Tumor group. In the cisplatin-treated group, p-Akt and p-mTOR levels were lower than those in the Tumor group, whereas Bax and cleaved caspase-3 levels were higher, and Bcl-2 levels were lower.

### 3.7. ODSW-CE Modulates Stress-Responsive and Redox-Associated Proteins in Tumor Tissues

**Figure 7:**
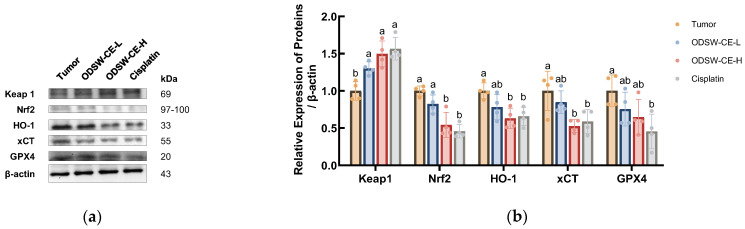
Effects of the ODSW-CE on the Nrf2/Keap1 antioxidant defense system and redox-related proteins in tumor tissues. Representative Western blot images showing the protein expression levels of Keap1, Nrf2, HO-1, xCT, and GPX4 in tumor tissues from each group (**a**). Relative protein expression levels normalized to β-actin (**b**). Data are presented as the mean ± SD (*n* = 4). For each protein, statistical comparisons were performed independently among groups. Groups with no lowercase letters in common are significantly different, whereas groups sharing at least one lowercase letter are not significantly different (*p* < 0.05), as determined by one-way ANOVA followed by Tukey’s multiple comparison test.

The results of the analysis of the expression of the Keap1/Nrf2 axis and redox-related proteins in tumor tissues are shown in Figure 7. In the Tumor group, Nrf2 expression was higher, while Keap1 expression was lower. Furthermore, HO-1, xCT, and GPX4 expression were higher in the Tumor group. Conversely, in the ODSW-CE-H-treated group, Nrf2 expression was lower, whereas Keap1 expression was higher compared with the Tumor group. The expression of HO-1, xCT, and GPX4 was also lower in the ODSW-CE-H-treated group compared with the Tumor group. In the cisplatin-treated group, Keap1 levels were higher than those in the Tumor group, whereas Nrf2, HO-1, xCT, and GPX4 levels were lower.

## 4. Discussion

Lung cancer remains one of the leading causes of mortality worldwide [1,2]. Despite advances in current treatment options, lung cancer continues to be associated with unclear clinical outcomes [1,2]. The overall efficacy of chemotherapy, targeted therapy, and immunotherapy is often limited by acquired resistance, heterogeneous responses, and toxicities associated with treatment [2,23]. These limitations have propelled an interest in adjunct strategies derived from natural products [2,14,23]. Among candidates of natural products, extracts derived from chili peppers have attracted attention because they contain carotenoids and other lipophilic phytochemicals with reported antioxidant, anti-inflammatory, and anticancer properties [24,25]. In our study, targeted HPLC-DAD analysis detected and quantified lutein and β-carotene in ODSW-CE (Figure 1). Comparative analysis showed that ODSW-CE contained relatively higher β-carotene and modestly higher rutin than extracts from conventionally cultivated peppers (Figure S1). However, several additional chromatographic peaks remained unidentified, suggesting that ODSW-CE contains other constituents that were not characterized by the present targeted analysis. Although lutein and β-carotene have been reported to exhibit anti-inflammatory and antioxidant activities [15,16,17,18], their individual contributions to the biological effects observed in the present study were not determined. Accordingly, subsequent analyses focused on the broader functional properties and biological activity of ODSW-CE at the extract level, rather than attributing its potential effects to individual carotenoids.

Preliminary comparison of the chili pepper extracts showed that ODSW-CE showed higher ABTS radical scavenging activity than the extracts obtained from conventionally and organically cultivated chili peppers (Figure S2). Subsequently, the antitumor activity of ODSW-CE was evaluated, which was characterized by reduced MTT-derived cellular metabolic activity (Figure 2a,b and Figure S3), impaired colony-forming capacity (Figure 2c,d), and reduced tumor burden in LLC1 tumor-bearing mice (Figure 3). Because the MTT assay reflects cellular reductive metabolic activity rather than directly measuring cell death, the reduction in the MTT signal was interpreted together with the colony formation and in vivo tumor growth results. Other extracts derived from *Capsicum annuum* have demonstrated similar antitumor activities [25,26,27]. In hepatocellular carcinoma models, the chili pepper leaf extract induced cytotoxicity, reduced the expression of pro-poly (ADP ribose) polymerase (PARP) and pro-caspase-3, suppressed signaling associated with glycolysis, and significantly inhibited tumor growth in a mouse model [26]. These observations suggest that the antitumor potential of phytochemicals, derived from peppers, go beyond a single, purified constituent. Pectic polysaccharides, isolated from sweet green pepper, reduced Ehrlich tumor growth in mice, decreased vascular endothelial growth factor (VEGF) expression and tumor vascular area, induced necrotic changes in tumor tissues, and inhibited the proliferation and viability of human mammary tumor cells [27]. By being highly selective for normal fibroblasts, ethanol extracts of seed and fruit of *Capsicum annuum* demonstrated cytotoxic activity against MCF7, A549, and C6 cancer cells [25]. The results of the present study suggest that the antitumor activity of ODSW-CE may result from the collective contributions of multiple constituents, including compounds not identified by the targeted HPLC-DAD analysis.

Inflammation is not merely a secondary consequence of tumor growth [3,28]. It actively promotes the establishment and progression of the tumor microenvironment [3,28]. In the present LLC1 tumor-bearing mouse model, inflammatory changes were observed at the hematological, biochemical, histological, and molecular levels (Figure 4 and Figure 5). NLR and MLR are commonly used indices of cancer-associated inflammation and immune imbalance [29,30]. These ratios reflect the relative predominance of neutrophil- and monocyte-associated inflammatory responses over lymphocyte-mediated antitumor immunity [29,30]. The increased NLR and MLR, together with alterations in white blood cell and neutrophil counts, indicate that LLC1 tumor growth was accompanied by systemic inflammatory activation. The reductions in NLR and MLR following ODSW-CE treatment suggest an improvement in systemic inflammatory status. The cytokine findings support this interpretation. TNF-α and IL-1β are key pro-inflammatory cytokines [3,28]. They amplify inflammatory signaling, recruit immune cells, and help maintain a microenvironment that favors tumors [28]. In the present study, ODSW-CE treatment was associated with lower plasma TNF-α and IL-1β concentrations and lower protein-normalized cytokine levels in lung tissue. Together with the hematological findings, these changes are consistent with attenuation of the overall tumor-associated inflammatory response. Histological observations were consistent with the biochemical findings. ODSW-CE-treated tumor tissues showed reduced hypercellular regions, suggesting attenuation of local histopathological alterations associated with tumor growth. In lung tissues, reduced inflammatory alterations further supported the mitigation of tumor-associated inflammatory changes beyond the primary tumor site.

These phenotypic changes were accompanied by decreased expression of TLR4, MyD88, p-IκB-α, p-NF-κB, COX-2, and TGF-β1. Mechanistically, activated TLR4 promotes MyD88-dependent signaling and recruits IL-1 receptor-associated kinase (IRAK) and TNF receptor-associated factor 6 (TRAF6) [31,32]. This activates the IκB kinase (IKK) complex, phosphorylates IκB-α, and leads to NF-κB activation [32]. Activated NF-κB increases the transcription of TNF-α, IL-1β, and COX-2 to reinforce an inflammatory cascade that promotes tumors [31]. Thus, the coordinated reductions in these signaling-related proteins and inflammatory indices suggest that the antitumor effects of ODSW-CE were associated with reduced TLR4/MyD88/NF-κB-related inflammatory signaling. Notably, cisplatin suppressed tumor growth, whereas the expression levels of TLR4, MyD88, p-IκB-α, p-NF-κB, COX-2, and TGF-β1 were higher than those in the Tumor group. This result suggests that the antitumor and inflammation-related responses to cisplatin may have occurred concurrently. Cisplatin treatment has been reported to increase extracellular high mobility group box 1 (HMGB1) release from murine LLC1 cells [33]. HMGB1 is a nuclear chromatin-associated protein that can function as a damage-associated molecular pattern following its extracellular release from damaged or dying cells [34]. In A549 lung cancer cells, cisplatin-induced HMGB1 secretion was dose-dependent and regulated by nuclear exportin 1 (XPO1)-mediated nuclear export [35]. The cytokine-active disulfide form of extracellular HMGB1 can bind myeloid differentiation factor 2 (MD-2) and activate TLR4-dependent NF-κB signaling [36]. HMGB1-mediated damage signaling may therefore have contributed, at least in part, to the elevated expression of inflammation-related proteins observed in cisplatin-treated tumors despite reduced tumor growth. In contrast, ODSW-CE suppressed tumor growth while reducing the expression of TLR4, MyD88, p-IκB-α, p-NF-κB, COX-2, and TGF-β1, indicating that its antitumor response was accompanied by attenuation of inflammation-related signaling. Consistent with the effects of ODSW-CE observed in our study, extracts derived from *Capsicum annuum* have also been reported to suppress tumor growth and to modulate signaling related to inflammation [26,27]. Chili pepper leaf extract suppressed tumor growth and reduced NF-κB expression in a hepatocellular carcinoma xenograft model [26]. Pectic polysaccharides from green sweet pepper also reduced tumor growth in tumor-bearing mice [27]. Taken together, these findings suggest that modulation of tumor-associated inflammatory signaling may contribute to the biological effects of ODSW-CE.

TLR4 signaling is not limited only to the NF-κB pathway [11] but is also functionally linked to the PI3K/Akt/mTOR axis, which regulates tumor cell growth and survival [11]. In some cellular contexts, TLR4 can also engage PI3K/Akt signaling [11]. This occurs through the p85 regulatory subunit of PI3K or adaptor molecules such as B-cell adapter for PI3K (BCAP) [37]. Thus, TLR4 may function as both an inflammatory receptor and an upstream regulator of survival signaling [11,37]. The PI3K/Akt/mTOR pathway supports protein synthesis, metabolic adaptation, cell proliferation, and resistance to apoptosis, which is closely associated with sustained tumor growth and therapeutic resistance [6]. In our study, ODSW-CE decreased the expression of PI3K, Akt, and mTOR (Figure 6) to suggest weakened survival signaling under stress conditions associated with tumors. Apoptosis is suppressed by PI3K/Akt signaling through Bcl-2 family proteins and other factors related to apoptosis [38]. Bcl-2 is an anti-apoptotic protein that preserves the integrity of the mitochondrial outer membrane and inhibits the release of cytochrome c [39]. In contrast, Bax is a representative protein that promotes apoptosis. It promotes the permeabilization of the mitochondrial outer membrane and the release of cytochrome c [40]. Therefore, the Bax/Bcl-2 ratio is widely considered an indicator of the intracellular balance that favors either survival or apoptosis [40,41]. Caspase-3 is a major executioner caspase that acts downstream of apoptotic signaling and completes the execution phase of apoptosis by cleaving multiple cytoplasmic and nuclear substrates [41]. In our study, ODSW-CE treatment changed the Bax/Bcl-2 balance and altered the markers associated with caspase-3. These changes indicate reduced resistance to apoptosis through ODSW-CE treatment. The findings of our study suggest that ODSW-CE weakened both inflammatory signaling associated with TLR4 and survival signaling dependent on the PI3K/Akt/mTOR pathway. Similar observations have been reported for extracts derived from peppers in other xenograft models [26]. Chili pepper leaf extract suppressed tumor growth in a Hep3B xenograft model [26]. It also reduced signaling associated with NF-κB and induced changes associated with caspase-3 [26]. Therefore, suppression of the TLR4/PI3K axis by ODSW-CE may contribute to changes associated with inflammation and growth observed in our study.

The suppression of survival signaling and induction of apoptosis were also associated with changes in redox defense-related proteins in tumor tissue [9,12,42,43]. Due to high metabolic activity and persistent oxidative stress, many tumor cells rely heavily on redox homeostasis to maintain their survival [9]. The Keap1/Nrf2 axis plays a central role in this process [12]. Under normal conditions, Nrf2 is restrained by Keap1 [12]. Under oxidative stress, Nrf2 is activated, translocates to the nucleus, and promotes the transcription of multiple defense genes through antioxidant response elements [43]. Nrf2-associated redox defense involves xCT, which contributes to cystine uptake and glutathione homeostasis, and GPX4, which removes lipid hydroperoxides [12,44]. These proteins are also recognized as key factors that maintain the tumor cell redox defense [43,45]. Sustained Nrf2/xCT/GPX4 signaling can protect tumor cells from oxidative damage and lipid peroxidation and thereby increase their resistance to oxidative stress-associated cell death [9,43]. In lung cancer, the aberrant activation of Keap1/Nrf2 signaling has been reported to support tumor growth and therapeutic resistance [43]. In the present study, ODSW-CE treatment increased Keap1 expression and decreased Nrf2, xCT, and GPX4 expression in LLC1 tumor tissue. These coordinated changes are consistent with attenuation of a redox defense axis that supports tumor adaptation to oxidative stress. Reduced xCT expression may lead to reduced cellular cystine uptake and reduced intracellular cysteine availability [43,45]. This could impair glutathione synthesis and diminish antioxidant buffering capacity [9,45]. Reduced GPX4 expression may indicate a diminished capacity to remove phospholipid hydroperoxides. However, the relationship between the observed changes in Nrf2, xCT, and GPX4 expression and ferroptotic cell death should be further validated using direct measures of glutathione depletion, lipid peroxidation, and ferroptosis-specific responses. Such analyses, together with evaluation of the corresponding responses in normal tissues, would help clarify the contribution of the Keap1/Nrf2/xCT/GPX4 axis to the antitumor response and determine whether the observed modulation is preferentially associated with tumor tissue. Nevertheless, the coordinated changes in protein expression observed in LLC1 tumor tissue are consistent with attenuation of the redox adaptation that supports tumor cell survival under oxidative stress.

## 5. Conclusions

Our study demonstrated the antitumor activity of ODSW-CE, an extract derived from chili pepper, in both LLC1 cells and mice bearing LLC1 tumors. We noted that ODSW-CE reduced MTT-derived cellular metabolic activity and the ability to form colonies in vitro and suppressed tumor growth in vivo. These effects were accompanied by lower TNF-α and IL-1β concentrations in plasma and reduced protein-normalized cytokine levels in lung tissue, together with improved histopathological features and decreased expression of proteins associated with TLR4/MyD88/NF-κB signaling. Collectively, these findings are consistent with attenuation of the overall tumor-associated inflammatory response. ODSW-CE was also associated with reduced PI3K, p-Akt, and p-mTOR expression and apoptosis-related changes, including increased Bax and cleaved caspase-3 expression together with decreased Bcl-2 expression. In addition, ODSW-CE altered tumor-associated redox defense-related proteins, as indicated by increased Keap1 and decreased Nrf2, xCT, and GPX4 expression. Taken together, our findings suggest that ODSW-CE may limit LLC1 tumor progression in association with coordinated changes in inflammatory signaling, tumor cell survival, apoptosis, and redox defense. As the present study evaluated ODSW-CE at the extract level, the relative contributions of lutein, β-carotene, and other constituents remain to be established through further chemical profiling and bioactivity-guided studies. Nevertheless, the present findings provide evidence for the antitumor potential of ODSW-CE as a biologically active chili pepper-derived extract.

## Figures and Tables

**Table 1:** Information of antibodies.

Antibody	Catalog No.	Dilution Rate	Company
TLR4	sc-293072	1:1000	Santa Cruz Biotechnology (Dallas, TX, USA)
MyD88	sc-74532		
Phosphorylated-inhibitor of κB-α (p-IκB-α)	sc-8404		
Phosphorylated-nuclear factor-κB (p-NF-κB)	sc-136548		
Cyclooxygenase-2 (COX-2)	ab15191		
Transforming growth factor-β1 (TGF-β1)	sc-130348		
p-mTOR	sc-293133		
p-Akt	sc-514032		
Bcl-2-associated X protein (Bax)	sc-7480		
B-cell lymphoma 2 (Bcl-2)	sc-7382		
kelch-like ECH-associated protein 1 (Keap1)	sc-514914		
β-actin	sc-69879	1:2000	
PI3K	#4255	1:1000	Cell Signaling Tech (Rosemont, IL, USA)
Caspase-3	#9662		
Nuclear factor erythroid-2-related factor 2 (Nrf2)	#20733		
Goat-anti-rabbit IgG	#7074	1:3000	
Heme oxygenase 1 (HO-1)	10701-1-ap	1:1000	Proteintech (Rosemont, IL, USA)
xCT	ab175186	1:1000	Abcam (Cambridge, UK)
Glutathione peroxidase (GPX)4	ab125066		
Goat-anti-mouse IgG	AP124P	1:3000	Sigma (St. Louis, MO, USA)

## Data Availability

The original contributions presented in this study are included in the article/Supplementary Materials. Further inquiries can be directed to the corresponding author.

## Supplementary Materials

The following supporting information can be downloaded at: https://www.mdpi.com/article/10.3390/antiox15070871/s1, Supplementary Materials and Methods: UPLC-DAD-APCI-QTOF/MS conditions; Figure S1: Quantification of lutein and β-carotene in chili pepper samples; Figure S2: Total phenolic content (TPC), total flavonoid content (TFC), and ABTS radical-scavenging activity of dried chili pepper samples; Figure S3: Effects of ODSW-CE, O-CE, and C-CE on LLC1 cell viability, as determined by the MTT assay; Figure S4: UPLC-DAD-APCI-QTOF-MS profiles of ODSW-CE; Table S1: Tentative annotation of major APCI-QTOF-MS features detected in ODSW-CE. The references cited in the Supplementary Materials are listed in the main reference list as Refs. [46,47,48,49,50].
