# Supplementary material for: Capsicum annuum Regulates Tumor Growth Through Modulation of TLR4/PI3K Signaling in a Lewis Lung Carcinoma Mouse Model
Source: Antioxidants (Basel). 2026 Jul 13;15(7):871. doi: 10.3390/antiox15070871 (PMC13404452; doi:10.3390/antiox15070871)
Supplement: Supplementary file 1 [file antioxidants-15-00871-s001.zip › antioxidants-4331092-supplementary.pdf]

Supplementary Materials

Capsicum annuum Regulates Tumor Growth Through Modulation of TLR4/PI3K Signaling in a Lewis Lung Carcinoma Mouse Model

Hye Ji Choi [1], Hyo Lim Lee [1], Yeong Hyeon Ju [1], Yu Mi Heo [1], Hwa Rang Na [1], Chae Eun Yoon[1], Young Hee Son[1], Do-Yoon Kim[1], Yu-Jin Kim[1], Hui-Seok Jeong [1], Seung-Hwan Park[2], Hyun-Jin Kim[1], and Ho Jin Heo [1,*]

1 Division of Applied Life Science (BK21), Institute of Agriculture and Life Science, Gyeongsang National University, Jinju 52828, Republic of Korea
2 Agriculture Research Center for Carbon Neutral and Healing, Gurye-gun 57607, Republic of Korea

* Correspondence: hjher@gnu.ac.kr; Tel.: +82-55-772-1907

Supplementary Materials

		PAGE
1	**Supplementary Materials and Methods**	*3*
	UPLC-DAD-APCI-QTOF/MS Conditions	
2	**Figure S1.** Quantification of lutein and β-carotene in chili pepper samples	*4*
3	**Figure S2.** Total phenolic content (TPC), total flavonoid content (TFC), and ABTS radical scavenging activity of chili pepper dry samples	*5*
4	**Figure S3.** Effects of ODSW-CE, O-CE, and C-CE on LLC1 cell viability, as determined by the MTT assay	*6*
5	**Figure S4.** UPLC-DAD-APCI-QTOF-MS profiles of ODSW-CE	*7*
6	**Table S1.** Tentative annotation of major APCI-QTOF-MS features detected in ODSW-CE	*8*

UPLC-DAD-APCI-QTOF/MS condition

Unbiased metabolomics analysis was performed using an ultra-performance liquid chromatography (UPLC) system (Waters, Milford, USA). The chromatographic separation was carried out using an ACQUITY UPLC HSS T3 column (100 mm × 2.1 mm, 1.8 μm, Waters) with a column temperature of 40 °C and a flow rate of 0.5 ml/min, where the mobile phase contained solvent A (water +0.1% formic acid) and solvent B (acetonitrile +0.1% formic acid). Metabolites were eluted using the following gradient elution conditions: 97% phase A for 0–5 min; 3–100% liner gradient phase B for 5 ~16 min; 100% phase B for 16–17 min; 100–3% reverse liner gradient phase B for 17~19 min; 97% Phase A for 19–25 min. The loading volume of each sample was 5 μl. The metabolites eluted from the column were detected by a high-resolution tandem mass spectrometer SYNAPT XS QTOF (Waters) in positive ion modes. For positive ion mode, the capillary voltage and the cone voltage were set at 2 kV and 40 V, respectively, and the APCI probe temperature was maintained at 450 °C. The primary scan ranged from 50–1200 m/z and the scanning time was 0.2 s. All the parent ions were fragmented using 20–40 eV. The information of all fragments were collected and

the time was 0.2 s. In the data acquisition process, the LE signal was gained every 3 s for real-time quality correction. Data acquisition and analysis were controlled by Waters UNIFI V1.71 software. The scan rang in MS and MS/MS modes were over a range of 100–1200 m/z.

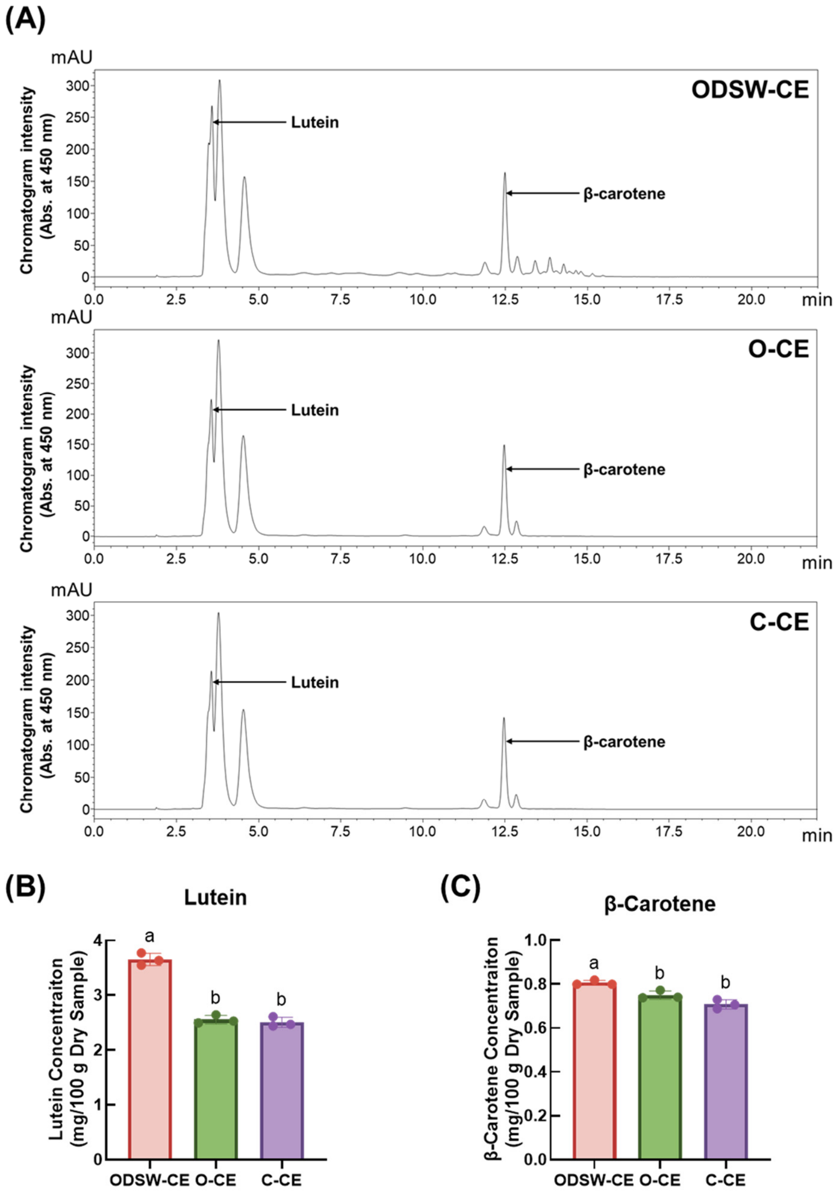

Figure S1. Quantification of lutein and β-carotene in chili pepper samples. **(A)** Representative HPLC chromatogram of lutein and β-carotene monitored at 450 nm. **(B)** Lutein concentration and **(C)** β-carotene concentration in chili pepper samples. Data are presented as the mean ± standard deviation

(SD) of three replicates (n = 3). Different letters above the bars indicate significant differences among samples according to one-way ANOVA followed by Tukey's multiple comparison test ($p < 0.05$). ODSW-CE: organic deep-sea water mineral-treated farming-*Capsicum annuum* extract, O-CE: organic farming-*Capsicum annuum* extract, C-CE: conventional farming-*Capsicum annuum* extract

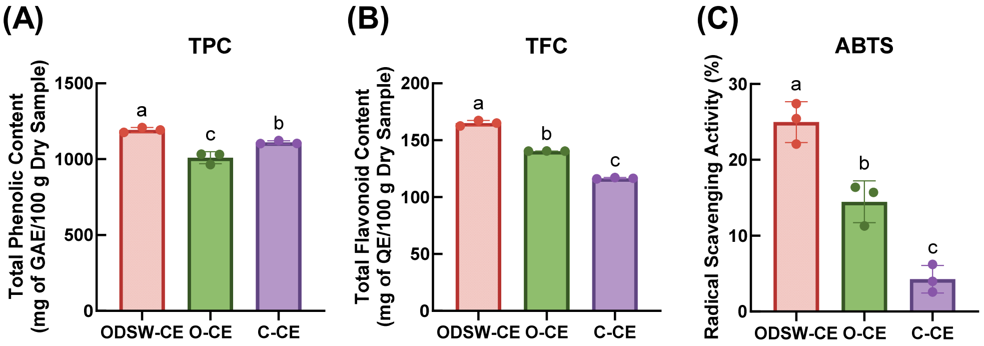

.

Figure S2. Total phenolic content (TPC), total flavonoid content (TFC), and ABTS radical scavenging activity of chili pepper dry samples. **(A)** TPC is expressed as mg gallic acid equivalents (GAE)/100 g dry samples, and **(B)** TFC is expressed as mg quercetin equivalents (QE)/100 g dry sample. **(C)** ABTS radical scavenging activity is expressed as a percentage (%). Values are presented as the mean ± SD (n = 3). Different letters above the bars indicate significant differences among samples, as determined by one-way ANOVA followed by Tukey's multiple comparison test ($p < 0.05$).

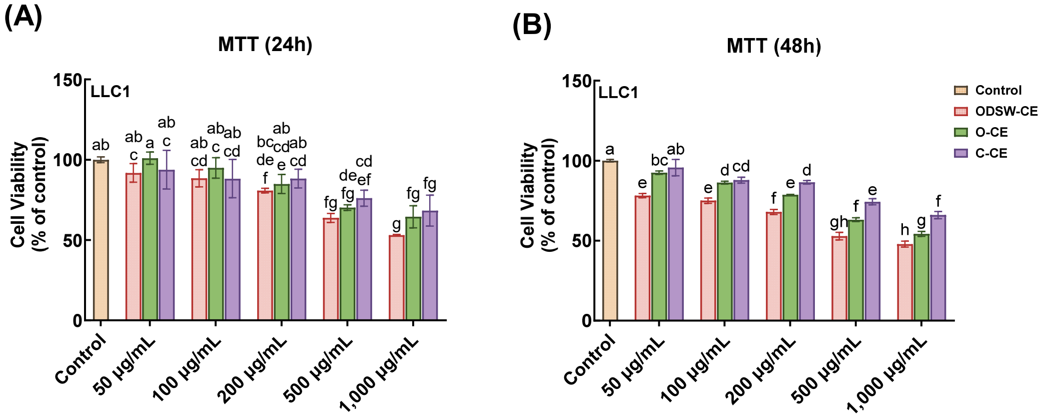

Figure S3. Effects of ODSW-CE, O-CE, and C-CE on LLC1 cell viability, as determined by the MTT assay. **(A)** LLC1 cells were treated with the indicated concentrations (50, 100, 200, 500, and 1,000 μg/mL) of each extract for 24 h, and cell viability was expressed as a percentage of the control. **(B)** LLC1 cells were treated under the same conditions for 48 h. Values are presented as the mean ± SD (n = 3). Different letters above the bars indicate significant differences among groups, as determined by one-way ANOVA followed by Tukey's multiple comparison test ($p < 0.05$).

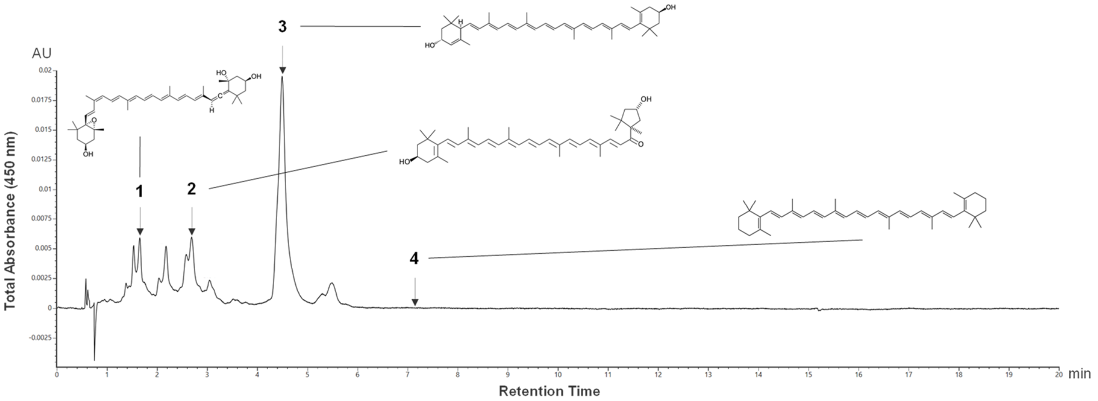

(A)

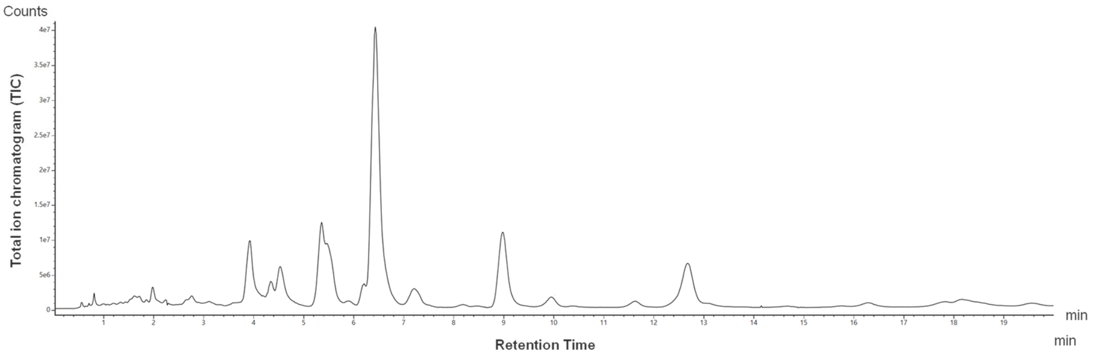

(B)

Figure S4. UPLC-DAD-APCI-QTOF-MS profiles of ODSW-CE. (**A**) UPLC-DAD chromatogram recorded at 450 nm with proposed structures for peaks 1–4. (**B**) APCI-QTOF-MS total ion chromatogram. Peak annotations were tentatively assigned based on accurate mass and fragmentation data, and the peak numbers correspond to Table S1.

Table S1. Tentative annotation of major APCI-QTOF-MS features detected in ODSW-CE

Peak	Retention Time (min)	Putative Compound	$[M+H]^+$ m/z	Fragment	Reference
**1**	1.67	Neoxanthin	601	583, 565, 547, 509, 491, 221	[46]
**2**	2.69	Capsanthin	585	567, 549, 492	[47]
**3**	4.49	lutein	569	551, 119, 175	[48]
**4**	7.12	β-carotene	537	536, 445	[49, 50]
